# The neurotrophic tyrosine kinase receptor TrkA and its ligand NGF are increased in squamous cell carcinomas of the lung

**DOI:** 10.1038/s41598-018-26408-2

**Published:** 2018-05-25

**Authors:** Fangfang Gao, Nathan Griffin, Sam Faulkner, Christopher W. Rowe, Lily Williams, Severine Roselli, Rick F. Thorne, Aysha Ferdoushi, Phillip Jobling, Marjorie M. Walker, Hubert Hondermarck

**Affiliations:** 1 School of Biomedical Sciences & Pharmacy, Faculty of Health and Medicine, University of Newcastle, Callaghan, NSW 2308 Australia; 2 Hunter Medical Research Institute, University of Newcastle, New Lambton, NSW 2305 Australia; 3 School of Medicine & Public Health, Faculty of Health and Medicine, University of Newcastle, Callaghan, NSW 2308 Australia

## Abstract

The neurotrophic tyrosine kinase receptor TrkA (NTRK1) and its ligand nerve growth factor (NGF) are emerging promoters of tumor progression. In lung cancer, drugs targeting TrkA are in clinical trials, but the clinicopathological significance of TrkA and NGF, as well as that of the precursor proNGF, the neurotrophin co-receptor p75^NTR^ and the proneurotrophin co-receptor sortilin, remains unclear. In the present study, analysis of these proteins was conducted by immunohistochemistry and digital quantification in a series of 204 lung cancers of different histological subtypes *versus* 121 normal lung tissues. TrkA immunoreactivity was increased in squamous cell carcinoma compared with benign and other malignant lung cancer histological subtypes (p < 0.0001). NGF and proNGF were also increased in squamous cell carcinoma, as well as in adenocarcinoma (p < 0.0001). In contrast, p75^NTR^ was increased across all lung cancer histological subtypes compared to normal lung (p < 0.0001). Sortilin was higher in adenocarcinoma and small cell carcinoma (p < 0.0001). Nerves in the tumor microenvironment were negative for TrkA, NGF, proNGF, p75^NTR^ and sortilin. In conclusion, these data suggest a preferential therapeutic value of targeting the NGF-TrkA axis in squamous cell carcinomas of the lung.

## Introduction

Lung cancer is the leading cause of cancer related death worldwide and its incidence is increasing^1^. Lung cancer histological subtypes are divided into two main categories: small cell lung cancers and non-small cell lung cancers (NSCLC). NSCLC represent the majority of lung cancer cases and include squamous cell carcinomas and adenocarcinomas. Despite extensive research, there are few clinically used biomarkers to help determine diagnosis, prognosis and treatment choice in lung cancer^1^. This is particularly problematic for NSCLC where correct identification of histological subtypes is essential to define the appropriate chemotherapeutic regimens. Therefore, the identification of pertinent biomarkers for diagnosis, stratification and therapeutic decision in lung cancer is necessary.

The neurotrophic tyrosine kinase receptor TrkA (NTRK1) and its ligand nerve growth factor (NGF) are essential to the development of the nervous system where they stimulate the outgrowth of sympathetic and sensory neurons^2^. Interestingly, TrkA and NGF are also expressed in several malignancies. In breast cancer, they participate in tumor cell proliferation and spreading *via* the activation of signalling pathways similar to those activated in neurons and including ERK, SRC and AKT^3^. Recent evidence in gastric^4^ and pancreatic^5^ cancers has shown that the NGF-TrkA signalling pathway is an essential and targetable stimulator of cancer progression. In lung cancer, rearrangements of TrkA have been shown to be oncogenic and are drug-sensitive^6^. TrkA is increasingly regarded as a therapeutic target in lung cancer and clinical trials of drugs against its tyrosine kinase activity are under way^7^. A previous study has shown that the expression of TrkA and NGF is higher in NSCLC^8^, but the distribution of TrkA and NGF in the different subtypes of lung cancer remains unclear. In addition, the expression of the other co-receptors for NGF^2^, the common neurotrophin receptor p75^NTR^ (also called NGFR or CD271), as well as that of the precursor for NGF (proNGF) and its receptor sortilin (a member of the Vacuolar Protein Sorting 10 protein - VPS10P - family of receptors) have not been elucidated. Despite early studies showing that neurotrophic growth factors are expressed in lung cancer^9^, the clinicopathological significance warrants clarification.

The present study aimed to clarify the expression and clinicopathological significance of TrkA, NGF, proNGF, p75^NTR^ and sortilin in lung cancer. The expression of these proteins was analysed by immunohistochemistry in a cohort of lung cancers versus normal lung tissues. We report an increased level of TrkA and NGF in squamous cell carcinomas, suggesting that drugs targeting the NGF-TrkA pathway in lung cancer should be used preferentially in this form of the disease.

## Results

**Figure 1:**
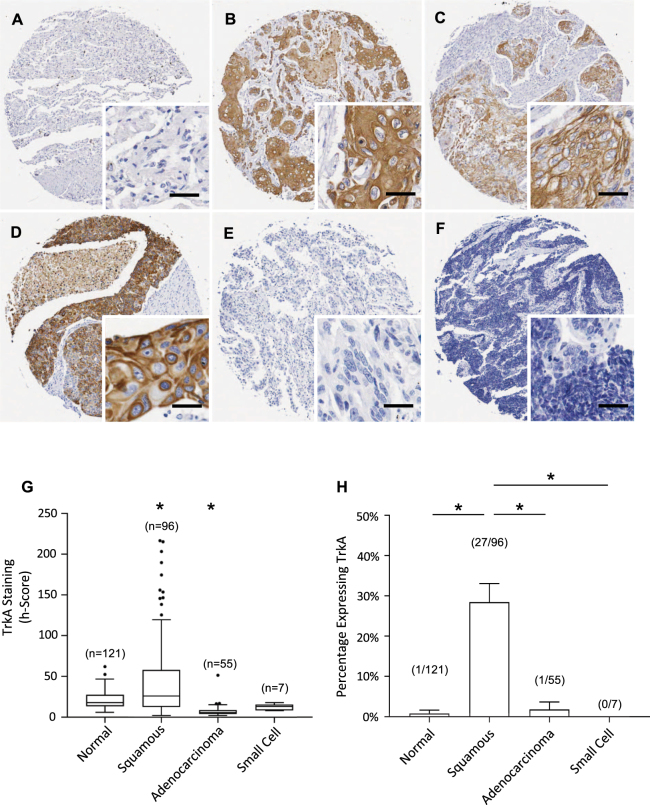
TrkA expression in lung cancers and normal lung tissues. (**A**–**F**) Immunohistochemical detection of TrkA, representative pictures are shown for normal tissue (**A**), squamous cell carcinoma (**B**–**D**), adenocarcinoma (**E**) and small cell cancer (**F**). Scale = 50 μm. (**G)** TrkA staining intensities were significantly higher in squamous cell carcinoma. Corresponding median h-scores are presented in Table 1. The box limits indicate the interquartile range (IQR) with the whiskers extending 1.5 times the IQR from the 25^th^ and 75^th^ percentiles (outliers are represented by dots) (^*^p < 0.0001 in multiple logistic regression model). (**H)** Proportion of tissues expressing TrkA receptor (binary h-score cutoff of 50) in normal lung tissue vs lung cancer subtypes. Squamous cell carcinoma was significantly higher than all other categories (p < 0.0001).

**Figure 2:**
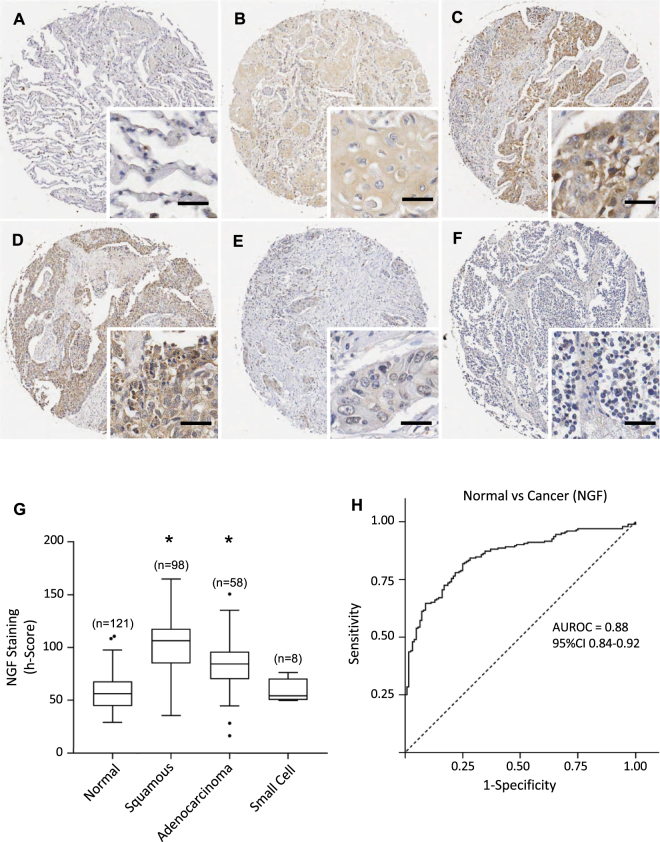
NGF expression in lung cancers and normal lung tissues. (**A**–**F**) Immunohistochemical detection of NGF, representative pictures are shown for normal tissue (**A**), squamous cell carcinoma (**B**–**D**), adenocarcinoma (**E**) and small cell cancer (**F**). Scale = 50 μm. (**G**) NGF staining intensities were significantly higher in squamous cell carcinoma and adenocarcinoma than in normal tissues. Corresponding median h-scores are presented in Table 1. The box limits indicate the interquartile range (IQR) with the whiskers extending 1.5 times the IQR from the 25^th^ and 75^th^ percentiles (outliers are represented by dots) (*p < 0.0001 in multiple logistic regression model). (**H**) ROC curve for NGF staining intensity level in lung cancers versus normal tissues. The area under the curve was 0.88 (95%CI 0.84 to 0.92).

**Figure 3:**
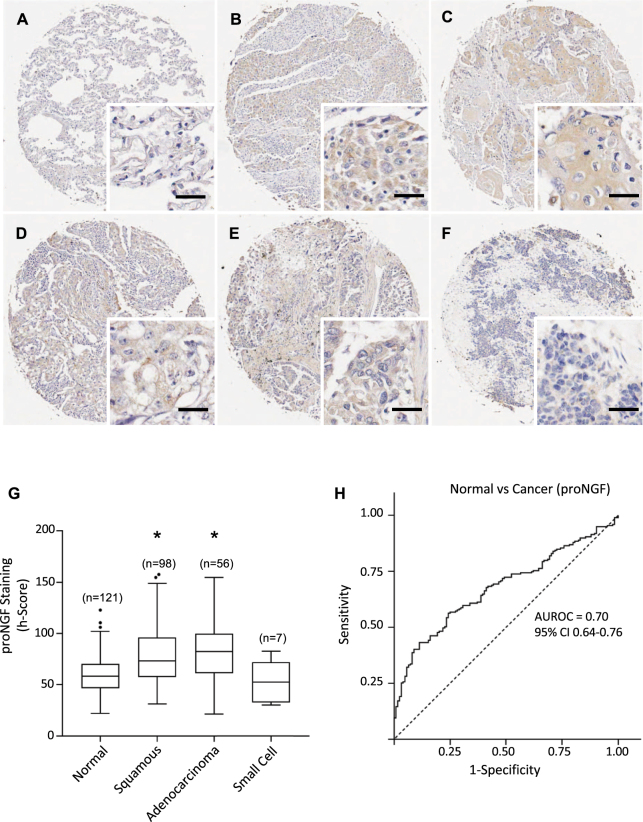
ProNGF expression in lung cancers and normal tissues. (**A**–**F**) Immunohistochemical detection of proNGF, representative pictures are shown for normal tissue (**A**), squamous cell carcinoma (**B**–**D**), adenocarcinoma (**E**) and small cell cancer (**F**). Scale = 50 μm. (**G**) ProNGF staining intensities were significantly higher in squamous cell carcinoma and adenocarcinoma than in normal tissues. Corresponding median h-scores are presented in Table 1. The box limits indicate the interquartile range (IQR) with the whiskers extending 1.5 times the IQR from the 25^th^ and 75^th^ percentiles (outliers are represented by dots) (*p < 0.0001 in multiple logistic regression model). (**H**) ROC curve for proNGF staining intensity level in lung cancers versus normal tissues. The area under the curve was 0.70 (95% CI 0.64 to 0.76).

**Figure 4:**
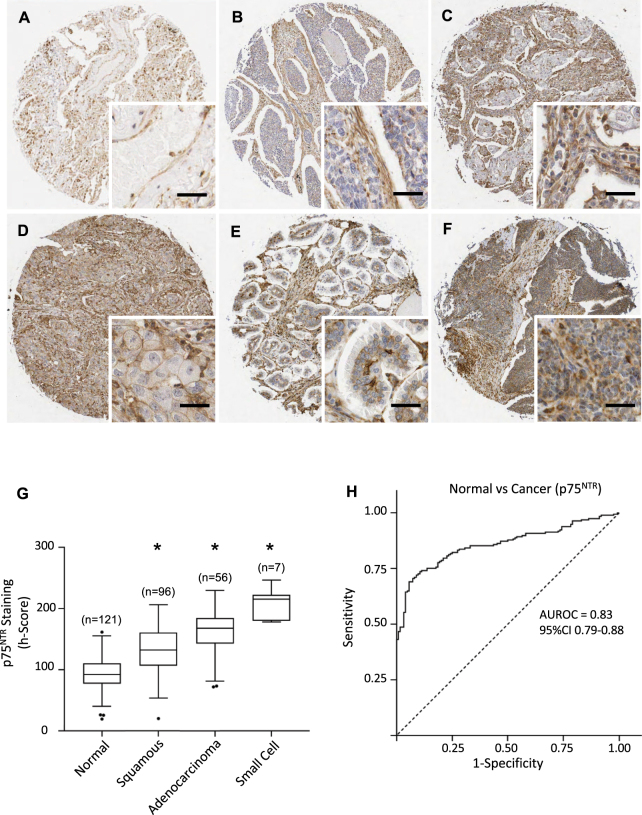
P75^NTR^ expression in lung cancers and normal lung tissues. (**A**–**F)** Immunohistochemical detection of p75^NTR^, representative pictures are shown for normal tissue (**A**), squamous cell carcinoma (**B**–**D**), adenocarcinoma (**E**) and small cell cancer (**F**). Scale = 50 μm. (**G**) p75^NTR^ staining intensities were significantly higher in squamous cell, adenocarcinoma and small cell cancers. Corresponding median h-scores are presented in Table 1. The box limits indicate the interquartile range (IQR) with the whiskers extending 1.5 times the IQR from the 25^th^ and 75^th^ percentiles (outliers are represented by dots) (*p < 0.0001 in multiple logistic regression model). (**H**) ROC curve for p75^NTR^ staining intensity level in lung cancers versus normal tissues. The area under the curve was 0.83 (95% CI 0.79 to 0.88).

**Figure 5:**
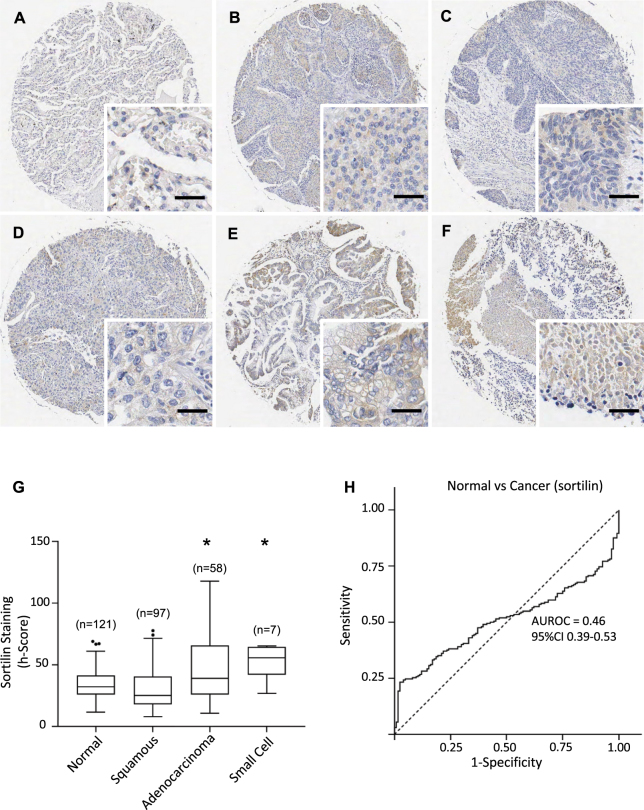
Sortilin expression in lung cancers and normal lung tissues. (**A**–**F)** Immunohistochemical detection of sortilin, representative pictures are shown for normal tissue (**A**), squamous cell carcinoma (**B**–**D**), adenocarcinoma (**E**) and small cell cancer (**F**). Scale = 50 μm. (**G**) Sortilin staining intensities were significantly higher in adenocarcinoma and small cell cancer. Corresponding median h-scores are presented in Table 1. The box limits indicate the interquartile range (IQR) with the whiskers extending 1.5 times the IQR from the 25^th^ and 75^th^ percentiles (outliers are represented by dots) (*p < 0.0001 in multiple logistic regression model). (**H**) ROC curve for sortilin staining intensity level in lung cancers versus normal tissues. The area under the curve was 0.46 (95%CI 0.39 to 0.53).

For all neurotrophins/receptors, representative pictures of immunohistochemical staining are shown in Figs 1–5 and quantification of staining intensities are presented in Table 1. Staining intensities (h-scores) are presented as medians (50^th^ centile value).

**Table 1:** Expression of NGF, proNGF, TrkA, p75^NTR^ and sortilin in lung cancers and association with clinicopathological parameters.

Parameter	NGF Intensity			proNGF Intensity			TrkA Intensity			sortilin Intensity			p75NTR Intensity		
	Median h-score	IQR	p-value	Median h-score	IQR	p-value	Median h-score	IQR	p-value	Median h-score	IQR	p-value	Median h-score	IQR	p-value
**Normal vs cancer**			**<0.0001**			**<0.0001**			**0.002**			0.43			**<0.0001**
Normal (n = 121)	57	46–70		58	47–70		18	13–26		32	26–41		92	81–110	
Cancer (n = 164)	95	76–112		76	58–96		13	6–33		30	20–48		147	116–176	
**Histological Type**			**0.0001**			**0.0001**			**0.0001**			**0.0001**			**0.0001**
Squamous (n = 98)	107	85–118		73	58–96		26	13–57		25	18–40		132	107–161	
Adenocarcinoma (n = 58)	84	72–98		82	65–98		6	4–9		40	26–66		164	141–184	
Small Cell (n = 8)	54	50–70		52	33–72		13	8–16		56	42–65		215	180–222	
**Gender**			0.04			0.28			**<0.0001**			**0.002**			**0.0005**
Male (n = 125)	99	80–115		74	58–95		16	7–39		27	19–44		138	112–171	
Female (n = 39)	84	70–108		83	59–109		6	4–13		42	27–58		168	147–189	
**Age (yrs)**			0.56			0.35			0.31			0.7			0.35
< 50 (n = 40)	94	72–108		72	55–97		16	8–39		30	22–49		154	121–177	
> 50 (n = 124)	96	76–114		76	59–96		11	6–29		31	19–48		141	114–175	
**Grade**			0.85			0.03			0.24			0.88			0.37
1 (n = 13)	98	84–117		96	89–120		19	10–138		26	18–46		141	82–160	
2 + 3 (n = 135)	97	78–114		74	59–95		14	6–34		29	19–46		142	116–172	
Missing (n = 16)	65	51–89		65	35–83		11	7–15		47	27–58		180	130–215	
**T stage**			0.88			0.55			0.63			0.71			0.8
T1/T2 (n = 133)	97	75–112		75	58–96		13	6–32		31	20–48		150	115–176	
T3/T4 (n = 31)	95	77–109		76	58–99		13	5–34		27	18–50		142	117–171	
**LN Status**			0.9			0.78			0.64			0.97			0.48
Negative (n = 70)	96	78–111		76	60–96		13	6–37		31	20–46		149	117–169	
Positive (n = 94)	95	75–112		74	56–96		13	5–31		29	20–49		145	116–178	
**Stage**			0.38			0.35			0.53			0.93			0.5
I + II (n = 120)	94	75–112		74	58–95		13	6–32		31	20–47		151	116–177	
III + IV (n = 44)	97	79–113		82	58–100		13	5–34		29	20–52		141	114–172	

Immunohistochemical staining were quantified and h-scores were used to compare protein expression levels. Group-levels medians (IQR, interquartile range) for h-score staining intensities are presented. Family-wise alpha significance level is 0.05/8 = 0.006 using the Wilcoxon Rank-Sum test (pairwise) or Kruskal-Wallis test (multiple comparisons). Statistically significant p-values are shown in bold.

### TrkA is increased in squamous cell lung carcinoma

Compared with normal lung (Fig. 1A), TrkA labelling was concentrated in cancer epithelial cells (Fig. 1B–F), with an increased staining intensity specifically in squamous cell carcinoma (Fig. 1B–D). Membrane staining was clearly observable. TrkA immunoreactivity was low in normal lung tissue (h-score 18), and lower in adenocarcinoma (h-score 6) (p < 0.0001); in squamous cell carcinoma, TrkA intensity (h-score 26) was significantly higher (p < 0.0001) (Fig. 1G, Table 1). This increase in h-score was driven by a subpopulation of squamous cell tumours that were strongly positive for TrkA, consistent with a binary receptor expression pattern of TrkA-present (h-score > 50) or TrkA-absent (h-score ≤ 50). When analysed by these parameters (Fig. 1H), 27/96 (28%) of squamous cell carcinomas expressed TrkA, compared to 1/55 (2%) of adenocarcinomas, 0/7 (0%) of small cell cancers and 1/121 normal tissues (<1%) (p < 0.0001), with 12/96 (13%) of squamous cell carcinomas showing very strong TrkA expression (h-score >100). Multivariate logistic regression modelling confirmed squamous pathology was significantly associated with increased TrkA h-score, when accounting for age and gender (Odds ratio (OR) 1.03, 1.01–1.04, p < 0.001). Given that TrkA was not expressed in most adenocarcinomas and small cell lung cancers, the area under the receiver-operating characteristic (AUROC) for the comparison between normal and cancer samples was only 0.39 (95%CI 0.32 to 0.46) (data not shown). No association was found between TrkA expression and age, grade, tumor size, stage, lymph node status.

### NGF and proNGF are increased in squamous cell carcinoma and adenocarcinoma

NGF immunoreactivity was observed at low levels in normal tissues (Fig. 2A), and was increased in cancer vs normal samples (Fig. 2B–F). NGF staining intensity (h-score) was significantly increased from 57 in normal to 95 in cancer samples (p < 0.0001) (Fig. 2G). Squamous cell carcinoma (Fig. 2B–D) and adenocarcinoma (Fig. 2E) presented with a NGF h-score of 107 and 84 respectively (p < 0.0001) (Fig. 2G, Table 1). Small cell cancers (Fig. 2F) displayed a lower level of NGF staining compared to other cancer subtypes (h-score 54) (Fig. 2G, Table 1) (p < 0.0001). Multivariate logistic regression modelling confirmed squamous cell carcinoma and adenocarcinoma were significantly associated with increased NGF h-score compared to benign pathology, when accounting for age and gender (OR 1.09 (1.06–1.12) and 1.08 (1.05–1.11) respectively, p < 0.001). The AUROC for the comparison between normal and cancer tissue was 0.88 (95%CI 0.84 to 0.92) (Fig. 2H).

ProNGF was also increased in malignant tissue (Fig. 3B–E) compared to normal lung tissue (Fig. 3A), but the differential in median h-score was less than NGF. Multivariate logistic regression modelling confirmed squamous cell carcinoma and adenocarcinoma were significantly associated with increased proNGF h-score compared to benign pathology, when accounting for age and gender (OR 1.04 (1.02–1.05) and OR 1.04 (1.03–1.06) respectively, p < 0.001) (Fig. 3G, Table 1). The AUROC for the comparison between normal and cancer samples was 0.70 (95%CI 0.64 to 0.76) (Fig. 3H).

For both NGF and proNGF, there was no association with age, grade, stage, tumor size, or lymph node invasion (Table 1).

### P75^NTR^ is increased in all lung cancer subtypes

Immunostaining for p75^NTR^ was observed in both epithelial and stromal cells of normal (Fig. 4A) and cancer samples (Fig. 4B–F). However, p75^NTR^ staining intensity was higher in cancer, with an h-score of 92 in normal compared to 147 in cancer (p < 0.0001) (Fig. 4G, Table 1). The increase in p75^NTR^ staining intensity occurred in all histological subtypes of lung cancer (squamous cell, adenocarcinoma, small cell) but was particularly strong in small cell carcinoma (median h-score of 215) (Fig. 4G, Table 1). Multivariate logistic regression modelling confirmed malignant pathology was significantly associated with increased p75^NTR^ h-score for squamous cell carcinoma (OR 1.04 (1.02–1.05), p < 0.001), adenocarcinoma (OR 1.07 (1.05–1.10 p < 0.001), when compared to benign pathology, when accounting for age and gender (p < 0.001). We were unable to fit a logistic regression model for small cell carcinoma vs benign tissue due to perfect separation of h-scores (Fig. 4G). The AUROC for the comparison of cancer *vs* normal was 0.83 (95%CI 0.79 to 0.88) (Fig. 4H). There was no association between p75^NTR^ staining and tumor size, grade, stage or lymph node status.

### Sortilin is increased in adenocarcinoma and small cell lung cancer

Immunostaining for sortilin was weak and found mainly in epithelial cells of both normal (Fig. 5A) and cancer samples (Fig. 5B–F). There was no difference between sortilin staining intensity in benign vs all lung cancer tissues with h-scores of 32 vs 30 respectively (p = 0.43). However, there was a higher level of sortilin staining intensity in adenocarcinoma (OR 1.05 (1.03–1.07, p < 0.001) and small cell (OR 1.18 (1.06–1.31, p = 0.002) when compared to benign pathology in a multivariate logistic regression model. (Fig. 5G, Table 1). The AUROC for comparison of cancer vs normal was 0.46 (95%CI 0.39 to 0.53) (Fig. 5H), confirming that sortilin expression is not significantly modified when comparing all lung cancers to normal tissues. In addition, there were no associations between sortilin expression and age, grade, stage, tumor size or lymph node invasion.

### Nerves in the tumor microenvironment of lung cancer do not express NGF, proNGF, TrkA, p75^NTR^ and sortilin

**Figure 6:**
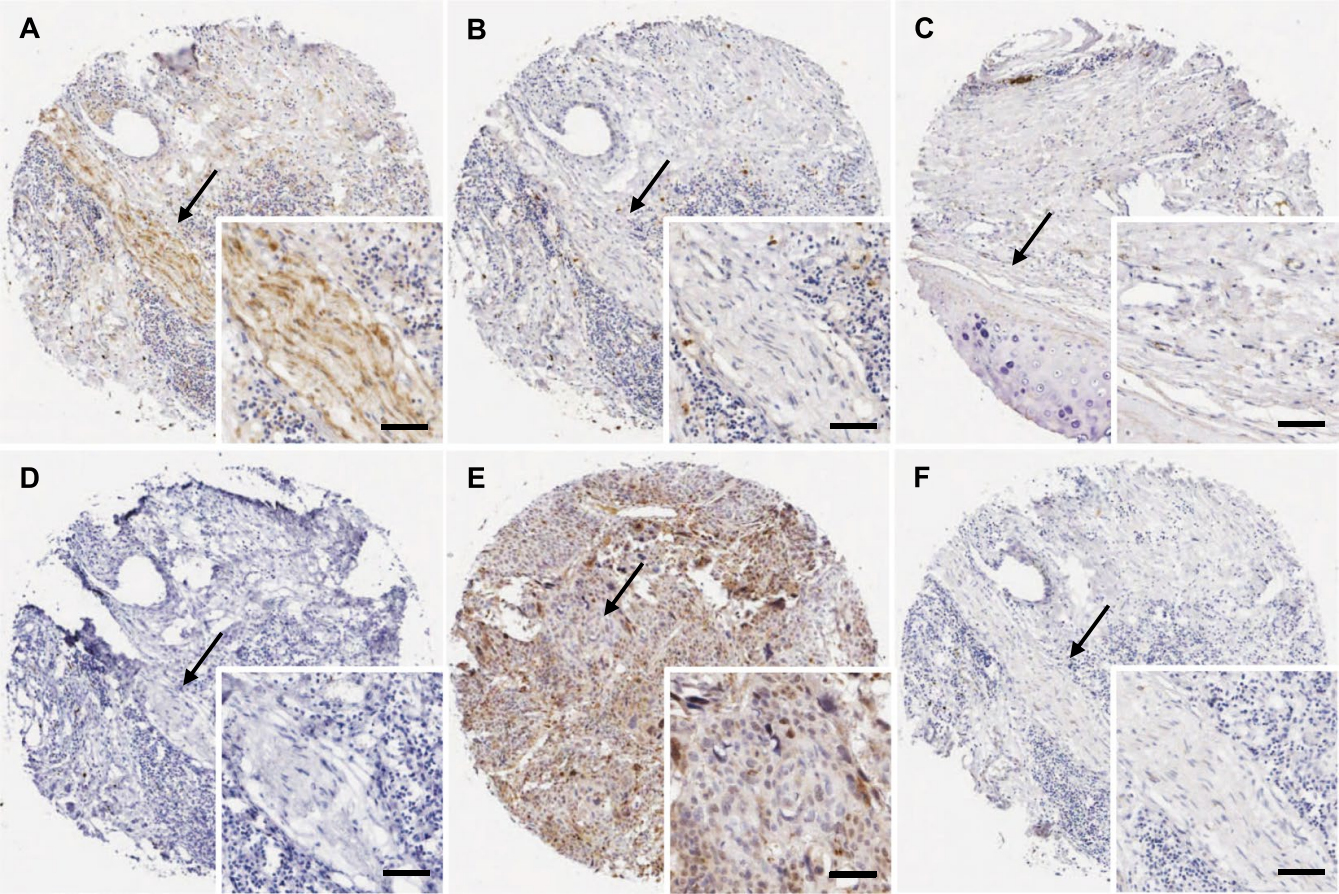
Nerves in the tumor microenvironment of lung cancer do not express NGF, proNGF, TrkA, p75^NTR^ and sortilin. (**A**) Immunohistochemical detection of the pan-neuronal marker PGP9.5 was used to detect nerves in lung cancers. The expression of NGF (**B**), proNGF (**C**), TrkA (**D**), p75^NTR^ (**E**) and sortilin (**F**) was not detected in serial sections. Black arrows indicate a nerve trunk composed of many axons. Scale = 25 μm.

The pan-neuronal marker PGP9.5 was used to detect nerves in the tumor microenvironment. Nerve trunks were occasionally detected in lung cancer (Fig. 6A), based on PGP9.5 positivity as well as typical nerve morphology. Serial sections were used and no labelling for NGF (Fig. 6B), proNGF (Fig. 6C), TrkA (Fig. 6D), p75^NTR^ (Fig. 6E) or sortilin (Fig. 6F) was detected. These data show that there is no expression of NGF, proNGF, TrkA, p75^NTR^ and sortilin in nerves which are present in the tumor microenvironment of lung cancer.

## Discussion

ProNGF and sortilin have not been described in lung cancers and there has been limited reports on the expression of NGF and its receptors TrkA and p75^NTR^^8,10,11^. Therefore, with only fragmentary data available, the clinicopathological significance remained unclear. To address this, we undertook a simultaneous investigation of the protein expression of proNGF, NGF, TrkA, p75^NTR^ and sortilin in the same cohort of lung cancers and normal lung tissues. Our results reveal that TrkA, NGF, proNGF, p75^NTR^ and sortilin are differently expressed across lung cancer histological subtypes, with TrkA and NGF most particularly increased in squamous cell carcinomas.

Prior to investigating protein levels by immunohistochemistry, data mining of gene expression in lung datasets available from The Cancer Genome Atlas (TCGA)^12^ using cBioportal^13^ was performed. Gene amplification and mRNA upregulation were detected at various frequencies in lung tumors: 6% for NGF, 14% for TrkA, 2% for p75^NTR^ and 4% for sortilin. However, discrepancies between mRNA and protein levels in tumors are now well documented. Global transcriptomic and proteomic analyses estimate that only 30%–60% of changes in protein levels can be explained by corresponding variations in mRNA^14,15^ and proteogenomic investigations in colorectal cancer have revealed that mRNA abundance does not reliably predict differences in tumoral protein abundance^16^. This emphasizes the importance of analysing the protein levels directly, in order to define new biomarkers and novel therapeutic targets in oncology.

The participation of TrkA and TrkA fusion proteins in lung cancer progression has been described, with Trk inhibitors undergoing clinical trials^6^. Our data shows a preferential expression of TrkA in squamous cell lung cancer, suggesting that Trk inhibitors should be used more specifically in this histological subtype of lung cancer. A previous investigation has reported an increase in TrkA and NGF in NSCLC and an association with tumor aggressiveness but not histological subtypes^8^. However, in this study staining intensities were visually determined, and no digital quantification assisted by a pathologist (as we have done here) was used, potentially leading to approximation in the quantification of expression levels. In addition, the comparison was done with normal adjacent tissues whereas true normal lung tissues were analysed in our study. Therefore, our study provides a refinement in terms of quantification of TrkA and NGF in lung cancer subtypes, highlighting a significant increase in both TrkA and NGF expression in squamous cell lung cancer. In contrast, as low expression level for TrkA were observed in adenocarcinoma and small cell cancer, it is unlikely that Trk inhibitors will produce any significant clinical impact in these tumors. Interestingly, NGF was also increased in squamous cell lung cancer and to a lesser extent in adenocarcinoma. This concomitant increased expression of both TrkA and its ligand NGF is suggestive of a NGF-mediated autocrine stimulation of squamous cell carcinoma. Similar autocrine stimulation of cancer cell growth via a proNGF/NGF autocrine loop involving TrkA has been described in breast cancer^17,18^ and may also apply to lung cancer. Although further functional investigations are warranted to test this hypothesis, our data reveals that TrkA and its ligand NGF are overexpressed in squamous cell lung cancer. This finding may have clinical ramifications, as humanized NGF blocking antibodies have been developed and are in clinical trials for the treatment of pain^19^; they may potentially be repurposed to inhibit the NGF-TrkA signaling axis in lung cancer.

In contrast to NGF, proNGF was only slightly increased in squamous cell lung cancer and adenocarcinoma. ProNGF processing into NGF requires protein convertases, such as furin or metalloproteases, and can occur both intracellularly or after secretion in the extracellular compartment^2^. In the nervous system, proNGF is the predominant protein form of NGF gene expression, with a higher presence of proNGF in comparison to NGF^20^. The regulatory mechanism that controls proNGF processing is poorly described in cancer, but our data suggest that proNGF is largely processed into NGF in squamous cell carcinomas of the lung. The limited differential in proNGF expression between normal and cancerous lung tissue is in line with the data obtained for its receptor sortilin. Sortilin was expressed at the same low levels in normal lung tissue, squamous cell carcinoma and was only higher in adenocarcinoma and small cell cancer. Sortilin has been reported in various cancer cell lines of different origins and its expression is associated to a poor prognosis in breast cancer where it participates in tumor cell migration and invasion^21^. In the squamous cell line A549, sortilin has been shown to participate in the transfer of exosomes in association with TrkB^22^, but our data have not highlighted any particular association between sortilin expression and clinicopathological parameters in any histopathological subtypes of lung cancers.

The neurotrophin receptor p75^NTR^ is expressed in a wide range of human tumors and has been shown to be a marker of cancer stem cells of both epithelial and mesenchymal origin^23^. However, the mechanism of p75^NTR^ activity in cancer cells is not fully elucidated and some studies in gastric^24^ and prostatic cancer^25,26^ have reported a tumor suppressor effect associated with p75^NTR^ suppression. In the present study, p75^NTR^ was expressed in normal lung tissues and overexpressed in all investigated lung cancer histological subtypes. The overexpression of p75^NTR^ was observed in epithelial cells as well as stromal cells. Interestingly p75^NTR^ has recently been shown to be a p53 inactivator^27^, and as such p75^NTR^ could actively participate in lung tumor growth, but further functional investigations are needed to explore this hypothesis.

Emerging evidence indicate the stimulatory role of nerves in tumor progression^28^. The nerve-cancer cell crosstalk involves the liberation of neurotransmitters and trophic factors to stimulate cancer cell growth and dissemination, while neurotrophic factors are released by cancer cells to attract nerve outgrowth in the tumor microenvironment^29^. In gastric cancer, NGF has been shown to activate cholinergic nerve-mediated signalling that stimulates the proliferation of stem cells^4^. In prostate cancer, sympathetic and parasympathetic nerves participate in the stimulation of tumor growth and metastasis^30^ and proNGF released by prostate cancer cells is a driver of neuronal outgrowth^31^. In lung cancer, autonomic nerve infiltration is associated with pathological risk grading and poor patient prognosis^32^, but the drivers of nerve infiltration have not been identified. Our study showing that there is no expression of the receptors TrkA, p75^NTR^ and sortilin in nerves infiltrated in the tumor microenvironment, suggest that NGF/proNGF are not involved in stimulating the growth of nerves in lung cancer.

Overall, this study highlights the overexpression of NGF, proNGF and their receptors TrkA, p75^NTR^ in lung cancer with a differential expression related to histological subtypes. A similar increased expression of these neurotrophins and receptors has already been observed in breast^17,18,33^ and thyroid^34,35^ cancer, suggesting that the upregulation these proteins is a common molecular feature in these cancers. Pharmacological inhibitors against TrkA^7^ and humanized anti-NGF antibodies^19^ have been developed and could therefore be used as therapeutic tools in breast, thyroid and lung cancers. The overexpression of TrkA in squamous cell carcinomas of the lung is of particular interest, given that TrkA inhibitors have entered clinical trials for the treatment of lung cancer^7^. In the nervous system, neurons responsive to NGF express TrkA and p75^NTR^ and the stimulation of these receptors by NGF induces a cascade of intracellular signalings including SRC, AKT, PI3K, ERK and NFkB. In lung cancer cells, TrkA tyrosine kinase inhibitors induce lung cancer cell growth arrest and apoptosis^36^. Based on the present findings, the biological effect of targeting TrkA and NGF in lung cancer should be revisited in the context of squamous cell carcinomas with more functional *in vitro* and *in vivo* animal models. From a clinical perspective, our data suggest that anti-TrkA therapies may be more effective in squamous cell lung cancer and could eventually be associated with NGF targeting.

## Material and Methods

### Lung tissue samples

High-density tumor microarrays (TMA) were obtained from US Biomax Inc. (Maryland, USA). The TMAs used (catalogue numbers: LC2086, LC2087 and BC041115) included a total of 204 lung cancers (of adenocarcinoma, squamous cell carcinoma, small cell carcinoma or other minor subtypes) and 121 normal lung tissues. The following clinicopathological information was available: patient age and sex, histological subtype, tumor size, grade, stage and lymph node status. No information on treatment and patient survival was available. US Biomax Inc. quality controls are described as follows. Each single tissue spot on every array slide was individually examined by pathologists certified according to WHO published standardizations of diagnosis, classification and pathological grade. For each specimen collected, informed consent was obtained from both hospital and individual. Discrete legal consent was obtained and the rights to hold research uses for any purpose or further commercialized uses were waived. The study was approved by the Human Research Ethic Committee of the University of Newcastle and all experiments were performed in accordance with relevant guidelines and regulations.

### Immunohistochemistry

Immunohistochemistry (IHC) was performed as previously described^34^. After deparaffinization and rehydration of the TMA slides following standard procedures, heat induced epitope retrieval was carried out in a low pH, citrate based antigen unmasking solution (Vector Laboratories, California, USA, catalogue number H-3300) using a decloaking chamber (Biocare, West Midlands, United Kingdom) at 95 °C for 20 min. IHC was then performed using an ImmPRESS^TM^ HRP IgG (Peroxidase) Polymer Detection Kit (Vector Laboratories, California, USA), as per the manufacturer’s recommendations. Briefly, after inactivation of endogenous peroxidases with 0.3% H_2_O_2_, and blocking with 2.5% horse serum, primary followed by secondary antibodies were applied to the sections and revealed with DAB Peroxidase (HRP) Substrate Kit (Vector Laboratories, California, USA, catalogue number SK-4100). The following primary antibodies were used at 1/500 dilution: anti-proNGF (#AB9040, Merck Millipore), anti-NGF (#ab52918, Abcam), anti-TrkA (#2508, Cell Signaling), anti-p75^NTR^ (#4201, Cell Signaling), anti-sortilin (#ANT-009, Alomone Labs), anti-PGP9.5 (#ab15503, Abcam). Finally, TMA slides were counterstained with hematoxylin (Gill’s formulation, Vector Laboratories, California, USA), dehydrated and cleared in xylene before mounting in Ultramount #4 mounting media (Thermo Fisher Scientific, Victoria, Australia). Negative controls, using isotype control antibodies, are shown in Supplementary Fig. 1.

### Digital quantification of IHC staining intensities

Quantification of staining intensities was performed as previously described^34^ using the Aperio AT2 scanner (Leica Biosystems, Victoria, Australia) and the Halo™ image analysis platform (Indica Labs, New Mexico, USA) under the supervision of a pathologist (MMW). Pixel intensity values were used to determine the h-scores for each core (index calculated as the sum of 3x% of pixels with strong staining +2x% of pixels with intermediate staining +1x% pixels with weak staining). Each core of the TMAs was investigated and the data were then submitted to statistical analysis.

### Statistical analysis

H-scores for NGF, proNGF, sortilin, TrkA and p75^NTR^ were analysed as continuous variables. Major lung cancer subtypes (164 cases of adenocarcinoma, squamous cell carcinoma, small cell cancer) were analysed and are presented in Table 1 and Figures, with other minor subtypes excluded from analysis (n = 43). For demographic (age and sex) and disease-specific (histopathology classes of benign, squamous, small cell and adenocarcinoma; tumour size, grade, stage; and nodal status) outcomes of interest, group level medians and interquartile ranges were compared with the Wilcoxon RankSum test, employing a Bonferroni correction for multiple pairwise comparisons (alpha at the family-wise 0.05 level = 0.05/8). All multiple-comparisons used the Kruskal-Wallis test due to unequal variances, with an adjusted multiple-comparison alpha threshold. Analyses were based on complete cases.

We explored the association between the subtypes of pathology and neurotrophin h-score by fitting multiple logistic regression models to subsets of the data. We considered each of squamous cell carcinoma, adenocarcinoma and small cell cancer separately, dichotomised against the benign tissue group. All models adjusted for age and gender.

The discriminative value of each neurotrophin or receptor H-score as a biomarker of lung malignancy was assessed with receiver-operating characteristic (ROC) curves, where a value of 0.5 indicates no difference, and a value of 1 signifies perfect discrimination. All analyses were performed using Stata (version 14.1, Statacorp, Texas USA). Additional graphics were created using GraphPad Prism 7 (California, USA).

## Electronic supplementary material

Supplementary Figure 1
