# Supplementary material for: The neurotrophic tyrosine kinase receptor TrkA and its ligand NGF are increased in squamous cell carcinomas of the lung
Source: Sci Rep. 2018 May 25;8:8135. doi: 10.1038/s41598-018-26408-2 (PMC5970205; doi:10.1038/s41598-018-26408-2)
Supplement: Supplementary file 1 — Supplementary Figure 1 [file 41598_2018_26408_MOESM1_ESM.pdf]

The neurotrophic tyrosine kinase receptor TrkA and its ligand NGF are increased in squamous cell carcinomas of the lung.

Fangfang Gao[1,2], Nathan Griffin[1,2], Sam Faulkner[1,2], Christopher W. Rowe[2,3], Lily Williams[1], Severine Roselli[1,2], Rick F. Thorne[2], Aysha Ferdoushi[1,2], Phillip Jobling[1,2], Marjorie M. Walker[2,3], Hubert Hondermarck[1,2,*]

[1]School of Biomedical Sciences & Pharmacy, Faculty of Health and Medicine, University of Newcastle, Callaghan NSW 2308, Australia.
[2]Hunter Medical Research Institute, University of Newcastle, New Lambton NSW 2305, Australia.
[3]School of Public Health & Medicine, Faculty of Health and Medicine, University of Newcastle, Callaghan NSW 2308, Australia.

Corresponding author: Hubert Hondermarck, School of Biomedical Sciences & Pharmacy, Life Sciences Building (LS3-35), University of Newcastle, Callaghan NSW 2308, Australia. Tel: +61 2492 18830. Email: hubert.hondermarck@newcastle.edu.au

Supplementary Figure 1: Negative controls for immunohistochemistry. A. Rabbit isotype control antibody was used in immunohistochemistry for NGF (ab52918, 1:200), TrkA (cs2508, 1:200), $p75^{NTR}$ (cs4201, 1:400) and sortilin (ANT009,1:500). B. Mouse isotype control antibody was used in immunohistochemistry for proNGF (6E10E7, 2.5μg/ml). Scale=25μm

A

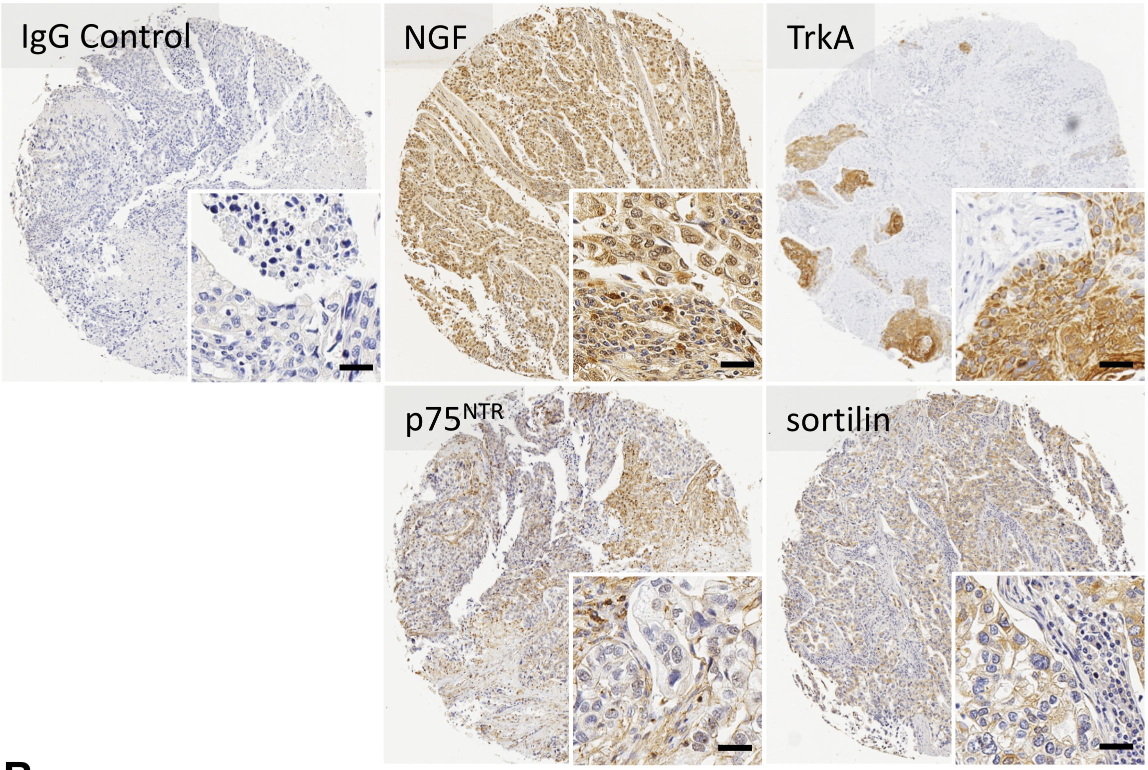

B

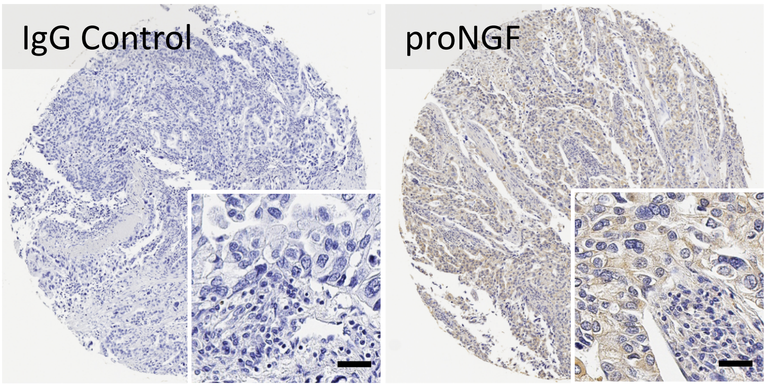

Supplementary Figure 1: Negative controls for immunohistochemistry. A. Rabbit isotype control antibody was used in immunohistochemistry for NGF (ab52918, 1:200), TrkA (cs2508, 1:200), p75^{NTR} (cs4201, 1:400) and sortilin (ANT009,1:500). B. Mouse isotype control antibody was used in immunohistochemistry for proNGF (6E10E7, 2.5µg/ml). Scale=25µm
